# *Momordica charantia* Ethanol Extract Attenuates H_2_O_2_-Induced Cell Death by Its Antioxidant and Anti-Apoptotic Properties in Human Neuroblastoma SK-N-MC Cells

**DOI:** 10.3390/nu10101368

**Published:** 2018-09-24

**Authors:** Kkot Byeol Kim, SeonAh Lee, Inhae Kang, Jung-Hee Kim

**Affiliations:** 1 Research Institute, Seoul Medical Center, Seoul 02053, Korea; naniccan03@gmail.com (K.B.K.); camui86@hanmail.net (S.L.); 2 Department of Food Science and Nutrition, Jeju National University, Jeju 63243, Korea; inhaek@jejunu.ac.kr; 3 Department of Neurosurgery, Seoul Medical Center, Seoul 02053, Korea

**Keywords:** *Momordica charantia*, oxidative stress, reactive oxygen species, neuroprotection, antioxidant, anti-apoptosis, SK-N-MC cells, neurodegenerative disease

## Abstract

Oxidative stress, which is induced by reactive oxygen species (ROS), causes cellular damage which contributes to the pathogenesis of neurodegenerative diseases. *Momordica charantia* (MC), a traditional medicinal plant, is known to have a variety of health benefits, such as antidiabetic, anti-inflammatory, and antioxidant effects. However, it is unknown whether MC has protective effects against oxidative stress-induced neuronal cell death. The aim of this study was to investigate the potential action of MC on oxidative stress induced by H_2_O_2_. First, we tested whether the pretreatment of *Momordica charantia* ethanol extract (MCEE) attenuates H_2_O_2_-induced cell death in human neuroblastoma SK-N-MC cells. MCEE pretreatment significantly improved cell viability and apoptosis that deteriorated by H_2_O_2_. Further, MCEE ameliorated the imbalance between intracellular ROS production and removal through the enhancement of the intracellular antioxidant system. Intriguingly, the inhibition of apoptosis was followed by the blockage of mitochondria-dependent cell death cascades and suppression of the phosphorylation of the mitogen-activated protein kinase signaling (MAPKs) pathway by MCEE. Taken together, MCEE was shown to be effective in protecting against H_2_O_2_-induced cell death through its antioxidant and anti-apoptotic properties.

## 1. Introduction

Neurodegenerative diseases (NDs), which are commonly associated with aging, are caused by the loss of progressive neuronal function [1,2,3]. NDs, including Alzheimer’s disease (AD), Parkinson’s disease (PD), Huntington’s disease (HD), and amyotrophic lateral sclerosis (ALS), are characterized by the progressive loss of cognition (dementia) and motor impairment (ataxia) [4,5,6]. These cognitive and motor impairments affect the life quality and life span of elderly individuals [7]. According to a World Health Organization (WHO) report (2005), the ND-related mortality rate will continue to increase to the second most common cause of death, following cardiovascular disease, by 2040 [8]. For this reason, identification of therapeutic agents for NDs is required for public health in modern society, especially considering the rapidly expanding elderly population [9].

One common pathway that has been proposed to be associated with the pathogenesis of aging-related diseases is the damage that results from the accumulation of reactive oxygen species (ROS) [4]. The oxidative stress induced by the accumulation of ROS plays a vital role in the onset and development of a number of diseases, especially NDs [10,11,12]. Compared with other body tissues, the brain is sensitive and vulnerable to oxidative stress damage due to its high oxygen demand, rich cellular lipids, and low levels of endogenous scavengers [13,14]. Therefore, protecting normal neuronal cells from damage or death by oxidative stress is a promising therapeutic approach for ND treatment, in terms of delayed disease progression and improved disease status [15]. In this respect, many studies have recently tried to find potential agents with neuroprotective effects.

Various natural products have long been used as traditional medicines for the treatment of NDs [16]. Many studies have suggested that neuronal cells are protected from oxidative stress-induced cell damage by polyphenolic compounds, which are obtained through the extraction of natural products [8]. *Momordica charantia* (MC), known as bitter melon or bitter gourd, is widely grown and usually consumed as an important medicinal plant in various regions of Asia, Africa, Central Asia, and South America [17,18]. MC contains several bioactive components, such as saponin, polysaccharide, vicine, polyphenols, vitamin C, and flavonoids [17,19]. Several studies have reported its therapeutic efficacy against various ailments via its antimicrobial, anticancer [20,21], anti-inflammatory [22], antioxidant [18,23], hypolipidemic [17,24], and antidiabetic [19,22,25] properties. In particular, it has been well-studied that MC can effectively ameliorate the symptoms of diabetes by several mechanisms, such as lowering the blood glucose level [26,27], stimulating the insulin secretion of β-cells [28], decreasing hepatic gluconeogenesis [29], and increasing the hepatic and muscle glycogen content [17,27]. However, it is unknown whether MC has protective effects against neuronal cell death due to oxidative stress.

The aim of this study was to evaluate the role of MC in regulating H_2_O_2_-induced oxidative stress for neuroprotection and to explore its potential mechanism of action. To accomplish this aim, we investigated the antioxidant and anti-apoptotic properties of MC in H_2_O_2_-induced human neuroblastoma SK-N-MC cells. Here, we present the first report that MC possesses biological activities to attenuate H_2_O_2_-induced cell death and improve the cellular antioxidant system. We also demonstrate that MC inhibits apoptosis by inhibiting the mitochondria-dependent apoptosis pathway and the mitogen-activated protein kinase signaling (MAPKs) pathway.

## 2. Materials and Methods

### 2.1. Preparation of 70% Ethanol Extract of Momordica Charantia (MCEE)

The dried fruits of *Momordica charantia* (MC) were purchased from KS Farm (Geumsan, Korea) in February 2017. A total of 4 g of dried MC powder was added to 70% ethanol (200 mL) and sonicated for 10 min. After primary incubation for 6 h at 150 rpm and 37 °C, the supernatant was removed, and a new portion of 70% ethanol (200 mL) was added and incubated a second time at 150 rpm and 37 °C for 18 h. After this, the primary and secondary incubation extracted solutions were combined and centrifuged at 3000 rpm for 3 min. The supernatant was then filtered through a 0.22 μm, PVDF syringe filter (Millipore, Bedford, MA, USA). The filtered solution was volatilized using a nitrogen generator. Finally, the obtained sample was dissolved in dimethyl sulfoxide (DMSO, Sigma, St. Louis, MO, USA) at a concentration of 200 mg/mL and stored in a −30 °C freezer.

### 2.2. Cell Culture and Treatment

The human neuroblastoma SK-N-MC cell line was obtained from the American Type Culture Collection (ATCC HTB-10, Manassas, VA, USA). The cells were grown in Eagle’s Minimum Essential Medium (EMEM, Gibco, BRL, Gaithersburg, MD, USA) supplemented with 10% fetal bovine serum (FBS, Sigma-Aldrich, St. Louis, MO, USA) and 1% anti-biotic/anti-mycotic (ABAM, Gibco-Invitrogen, Grand Island, NY, USA), and the cultures were maintained in a humidified incubator at 37 °C in an atmosphere of 5% CO_2_ and 95% air. The cell culture medium was changed every two days. When the cells were about 90% confluent, they were washed with PBS, detached with 0.25% trypsin EDTA (Gibco, BRL, Gaithersburg, MD, USA), resuspended, and subcultured onto plates at an appropriate density according to each experimental scale. Unless stated otherwise, cells were pretreated with various concentrations (5, 10, and 20 μg/mL) of MCEE for 24 h and then exposed to H_2_O_2_ (500 μM) for 4 h.

### 2.3. Cell Viability and Cytotoxicity

Cell viability was measured using the Cell Counting Kit (CCK)-8 assay (Dojindo, Tokyo, Japan). Briefly, SK-N-MC cells (1 × 10^4^ cells/well) were seeded in a 96-well plate. After 24 h of incubation, cells were pretreated with different concentrations of MCEE (5, 10, and 20 μg/mL) for 24 h, and later, 500 μM H_2_O_2_ was added for 4 h. After the treatment, the CCK-8 assay reagent was added to the culture media and incubated for 2 h. Absorbance was read at 450 nm on a microplate reader (Sunrise, Tecan, Grodig, Austria).

Cell cytotoxicity was measured using the LDH Cytotoxicity Detection Kit (Takara, Shuzo Shiga, Japan). Cells were plated at a density of 1 × 10^4^ cells/well in 96-well plates. In accordance with the manufacturer’s instructions, the supernatants were collected after treatment and incubated with the reagent mixture. After adding 1 N HCl to each well, absorbance was measured at about 490 nm on a microplate reader (Sunrise, Tecan, Grodig, Austria).

### 2.4. Hoechst 33342 Staining

Hoechst 33342 (Invitrogen Co, Carlsbad, CA, USA) staining was used to detect changes in the nuclei of apoptotic cells. SK-N-MC cells were plated at a density of 2 × 10^5^ cells/well in six-well plates and treated as described above. After the treatment, the culture medium was removed, and the cells were fixed with 4% paraformaldehyde (Sigma-Aldrich, St. Louis, MO, USA) for 10 min at room temperature. The fixed cells were incubated with Hoechst 33342 (final concentration 5 μg/mL) for 10 min at room temperature and subsequently washed with PBS. Then, the cells were observed under a fluorescence microscope (IX81, Olympus Corp., Tokyo, Japan) at 400× magnification.

### 2.5. Annexin V-FITC and PI Double-Staining Assay

The apoptotic and necrotic cells were evaluated and quantified by the FITC Annexin V Apoptosis Detection Kit I (BD Bioscience, San Jose, CA, USA) in accordance with the manufacturer’s instructions. In brief, after treatment, the cells were washed with ice-cold PBS, collected by centrifugation, and then resuspended in binding buffer. Five microliters of Annexin V-FITC and 5 μL of propidium iodide (PI) were added and incubated for 15 min at room temperature in the dark. Cells were analyzed using flow cytometry (FACSCalibur, Becton Dickinson, San Jose, CA, USA).

### 2.6. Measurement of Intracellular ROS

The intracellular ROS levels were determined with a DCFH-DA fluorescent probe (Sigma-Aldrich, St. Louis, MO, USA). SK-N-MC cells (1 × 10^5^ cells/well) were pretreated with varying concentrations of MCEE (5, 10, and 20 μg/mL) for 24 h and then 50 μM DCFH-DA for 30 min before being incubated with 500 μM H_2_O_2_ for 1 h. The cells were washed with PBS and the fluorescent compound was detected by a fluorescence microplate reader (Infinite M200, TECAN, Grodig, Austria) at the Ex/Em of 485 nm/530 nm and flow cytometry (FACSCallibur, Becton Dickinson).

### 2.7. Measurement of Superoxide Dismutase (SOD), Reduced Glutathione (GSH), and Malondialdehyde (MDA)

The SK-N-MC cells were plated at a density of 2 × 10^5^ cells/well in six-well plates and treated as described above. The protein concentration was determined with the BCA protein assay kit with BSA (Thermo Scientific, Rockford, IL, USA). SOD activity and MDA level (SOD assay kit and TBARS assay kit, Cell Biolabs, San Diego, CA, USA) were measured at wavelengths of 450 and 532 nm, respectively, in a microplate reader. The GSH level (GSH/GSSG ratio detection assay kit, Abcam, Cambridge, UK) was detected at the Ex/Em of 490 nm/520 nm.

### 2.8. Measurement of Mitochondrial Membrane Potential (MMP, ΔΨ_m_)

The mitochondrial membrane potential (MMP, ΔΨ_m_) was determined using a Flow CytometryMitochondrial Membrane Potential Detection Kit (BD Bioscience, San Jose, CA, USA). In accordance with the manufacturer’s instructions, the culture medium was removed after treatment, and the cells were incubated with JC-1 stain solution for 15 min at 37 °C. Then, the cells were washed and analyzed using flow cytometry (FACSCalibur, Becton Dickinson).

### 2.9. Total RNA Extraction and Quantitative Real-Time Polymerase Chain Reaction

Total RNA from SK-N-MC cells was extracted using the TRIZOL reagent (Invitrogen Co, Carlsbad, CA, USA). RNA concentrations were measured by a NanoDrop ND-2000 spectrophotometer (Thermo Fisher Scientific, Wilmington, DE, USA), and reverse transcription using the PrimeScript RT Master Mix (TaKaRa, Shuzo Shiga, Japan), in accordance with the manufacturer’s instructions. The mRNA expressions of HO-1 and glyceraldehyde 3-phosphate dehydrogenase (GAPDH) were measured using a LightCycler 480 system (Roche diagnostics Nederland BV) and a SYBR Premix Ex Tap TM kit (TaKaRa, Shuzo Shiga, Japan), in accordance with the manufacturer’s instructions. The following primers were used: heme oxidase (HO)-1 (Accession No. JF323038.1), L, 5′-GGC AGA GGG TGA TAG AAG AGG-3′ and R, 5′-AGC TCC TGC AAC TCC TCA AA-3′ and GAPDH (Accession No. BC025925.1), F, 5′-GCC CAA TAC GAC CAA ATC C -3′, and R, 5′-AGG CAC ATC GCT CAG ACA C-3′.

### 2.10. Western Blot Analysis

The cultures were washed with PBS and then incubated with RIPA lysis buffer (Sigma-Aldrich) containing protease and phosphatase inhibitor cocktails (Sigma-Aldrich). The protein concentration was measured by the BCA protein assay. The same amount of protein (20 μg/lane) was added to each lane to conduct the Western Blot Analysis. The samples were separated by 7.5–15% SDS-PAGE gel followed by transfer to a PVDF membrane (Merck Millipore, Darmstadt, Germany). After transfer, they were blocked for 1 h with 5% skim milk (Sigma-Aldrich) in TBS-T buffer. The membranes were incubated with primary antibodies against HO-1 (#5853), Bcl2 (#2872), Bax (#2772), cytochrome c (#4280), caspase-9 (#9502), caspase-3 (#9665), PARP (poly (ADP-ribose) polymerase, #9532), JNK (#9252), p-JNK (#9251), p38 (#8690), p-p38 (#9211), ERK1/2 (#9102), p-ERK1/2 (#9101), and GAPDH (#2118) overnight at 4 °C. After washing, the HRP-conjugated secondary antibody (anti-rabbit IgG, #7074) was diluted 1:2000 and reacted at room temperature for 1 h. All primary and secondary antibodies were purchased from Cell Signaling Technology (Boston, MA, USA). After washing three times with TBS-T, the ECL-Western blotting substrate (Thermo Scientific) was treated and the results were confirmed. The densities of the protein bands were quantified using Bio-1D imaging software (Vilber Lourmat, Marne-Ia-Vallee, France).

### 2.11. Statistical Analyses

All data are expressed as means ± standard deviation (S.D.). Statistical analyses were included t-tests and one-way ANOVAs using the statistical package SPSS software 20.0 (SPSS, Chicago, IL, USA). The test results were considered statistically significant when *p* < 0.05.

## 3. Results

### 3.1. Effect of the MCEE on H_2_O_2_-Induced Cytotoxicity in SK-N-MC Cells

**Figure 1:**
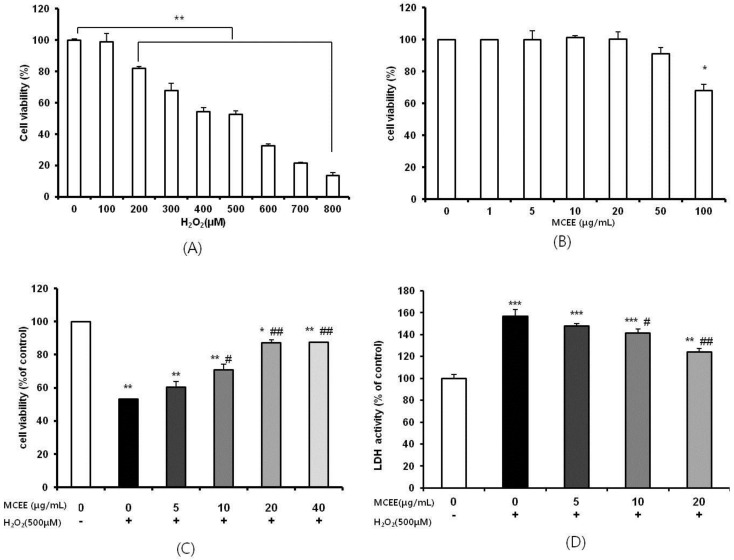

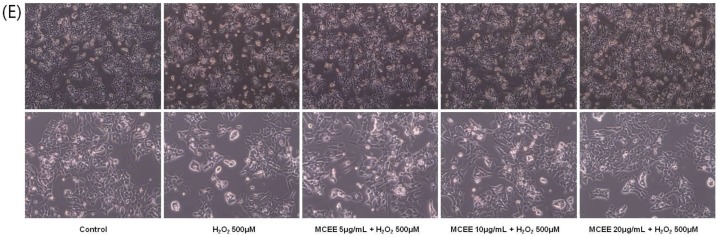
Effects of the *Momordica charantia* Ethanol Extract (MCEE) on the cell viability of and the cytotoxicity in SK-N-MC cells treated with H_2_O_2_. The cell viability was measured with the CCK-8 assay. SK-N-MC cells were treated with H_2_O_2_ (100–800 μM) (**A**) and the MCEE (1–100 μg/mL) (**B**) for 24 h. (**C**) Cells were pretreated with the MCEE (5–40 μg/mL) for 24 h and then treated with H_2_O_2_ (500 μM) for 4 h. (**D**) Cytotoxicity was determined with a lactase dehydrogenase (LDH) activity assay. (**E**) The morphological change of SK-N-MC cells was observed using a microscope (magnification 40× (upper)/100× (lower)). The cell viability was calculated as a percentage (%) of the control (**A**–**D**) and expressed as the means ± standard deviation (S.D.) (*n* = 3). * *p* < 0.05; ** *p* < 0.01; *** *p* < 0.001 compared with the untreated control cells; # *p* < 0.05; ## *p* < 0.01 compared with only H_2_O_2_-treated cells. +, treatment; -, non-treatment.

It is well-recognized that the human neuroblastoma SK-N-MC cell, which is induced by oxidative stress by H_2_O_2_, is a good in vitro model of NDs due to its high stability and homogeneity [1,30]. Exposure of the cells to H_2_O_2_ reduced cell viability dose-dependently (Figure 1A). Treatment with up to 50 μg/mL of MCEE was not associated with any significant changes to the cell viability of SK-N-MC cells (Figure 1B). To gain insight into whether MCEE has neuroprotective effects against H_2_O_2_, cells were pretreated with MCEE for 24 h, and then exposed to 500 μM H_2_O_2_ for 4 h (Figure 1C). While the cells exposed to 500 μM H_2_O_2_ for 4 h showed a remarkable reduction in cell viability (52.99 ± 0.08% compared with the control), pretreatment with different concentrations of MCEE (5, 10, 20, and 40 μg/mL) increased the cell viability in a dose-dependent manner. The cell viability significantly increased to 60.33% ± 3.41%, 70.75% ± 3.47%, 87.02% ± 1.98%, and 87.39% ± 0.27%, respectively, compared with the control group. The cell viability percentages at 20 μg/mL and 40 μg/mL were similar. Thus, 5, 10, and 20 μg/mL of MCEE were used for the remainder of the studies to further investigate the neuroprotective mechanisms of MCEE. These effects of MCEE were also observed in cell morphological change (Figure 1E).

The effect of MCEE on the release of lactate dehydrogenase (LDH), which is known to be an indicator of cell death [10,11], was evaluated in the culture medium and quantified under the same experimental conditions. As shown in Figure 1D, LDH release in the H_2_O_2_-stimulated group increased to 156.87 ± 6.07% of the control group. However, pretreatment with different concentrations of MCEE (5, 10, and 20 μg/mL) decreased the LDH release to 147.69% ± 2.31%, 141.54% ± 3.53%, and 123.84% ± 3.53% of the control group, respectively.

### 3.2. MCEE Decreased Intracellular ROS Produced by H_2_O_2_

**Figure 2:**
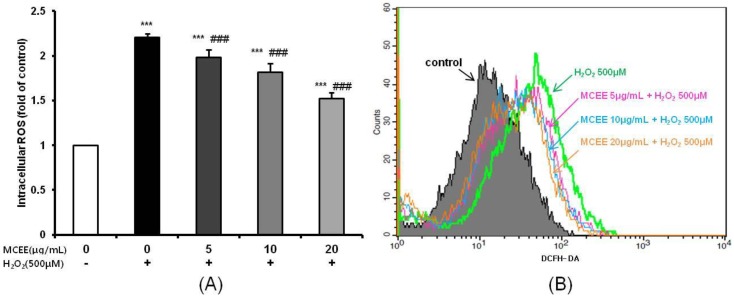
The inhibitory effects of MCEE on H_2_O_2_-induced intracellular reactive oxygen species (ROS) production in SK-N-MC cells. The intracellular ROS production was determined by the DCFH-DA method using a fluorescence microplate reader (**A**) and flow cytometry (**B**). The SK-N-MC cells were incubated with MCEE for 24 h, then treated with H_2_O_2_ for 1 h. The results are expressed as the means ± S.D. (*n* = 3). *** *p* < 0.001 compared with the untreated control cells, ### *p* < 0.001 compared with only H_2_O_2_-treated cells.

Next, we investigated the ability of MCEE to counteract intracellular ROS production, which is closely related to cell damage and death, by using DCFH-DA, a fluorescent ROS indicator. As shown in Figure 2, intracellular ROS levels in the H_2_O_2_-treated group were about 2.21 ± 0.04 fold higher than in the control group. In contrast, MCEE (5, 10, and 20 μg/mL) significantly diminished ROS generation in a dose-dependent manner (1.98 ± 0.09, 1.81 ± 0.10, and 1.52 ± 0.07 fold of the control group, respectively).

### 3.3. Effect of MCEE on Antioxidant Properties

**Figure 3:**
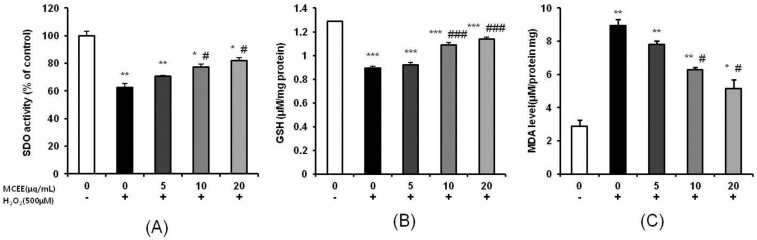
Effects of MCEE on (**A**) superoxide dismutase (SOD) activity, (**B**) reduced glutathione (GSH), and (**C**) malondialdehyde (MDA) levels in H_2_O_2_-treated cells. Cells were pretreated with the MCEE for 24 h and then treated with H_2_O_2_ for 4 h. Data are shown as means ± S.D. (*n* = 3). * *p* < 0.05; ** *p* < 0.01; *** *p* < 0.001 compared with the untreated control cells, # *p* < 0.05; ### *p* < 0.001 compared with only H_2_O_2_-treated cells.

It is known that components of the intracellular antioxidant defense system, such as SOD and GSH, play important roles in controlling the balance between the production and removal of ROS. As shown in Figure 3A,B, treatment with 500 μM H_2_O_2_ for 4 h caused decreases in the activity of SOD and level of GSH by 37.3% and 31.6%, respectively. In contrast, pretreatment with MCEE markedly increased SOD activity and the GSH level. In addition, MDA, which is one of the most important products of membrane lipid peroxidation and is widely used as a biomarker of oxidative stress [31,32], significantly increased by 310.0% in the H_2_O_2_-treated group. However, pretreatment of MCEE significantly attenuated the MDA level in a dose-dependent manner (Figure 3C).

**Figure 4:**
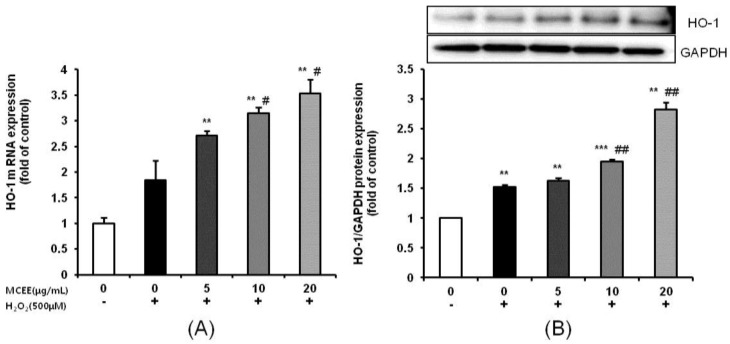
Effects of the MCEE on HO-1 expression in H_2_O_2_-induced SK-N-MC cells. Cells were pretreated with the MCEE for 24 h and then treated with H_2_O_2_ for 4 h. (**A**) The HO-1 mRNA expression level was quantified by real-time PCR analysis. The HO-1 mRNA expression level was normalized to the GAPDH expression level, which was used as an internal reference. (**B**) HO-1 protein expression was confirmed by Western blot analysis. The normalization of HO-1 used GAPDH. The results are expressed as means ± S.D. (*n* = 3). ** *p* < 0.01; *** *p* < 0.001 compared with the untreated control cells, # *p* < 0.05; ## *p* < 0.01 compared with only H_2_O_2_-treated cells. GAPDH, glyceraldehyde 3-phosphate dehydrogenase.

Next, we confirmed MCEE’s antioxidant function by investigating the mRNA and protein expression levels of HO-1, which is regarded as a key molecule for the maintenance of antioxidant homeostasis [33]. Treatment with 500 μM H_2_O_2_ for 4 h increased the mRNA and protein expression levels of HO-1. When the cells were pretreated with MCEE for 24 h before being exposed to H_2_O_2_ for 4 h, HO-1 mRNA and protein expression levels were dose-dependently increased (Figure 4). These results collectively demonstrate that (1) MCEE reduces H_2_O_2_-induced cytotoxicity and ROS levels, and (2) MCEE increases antioxidant levels.

### 3.4. MCEE Reduced H_2_O_2_-Induced Apoptotic Cell Death

**Figure 5:**
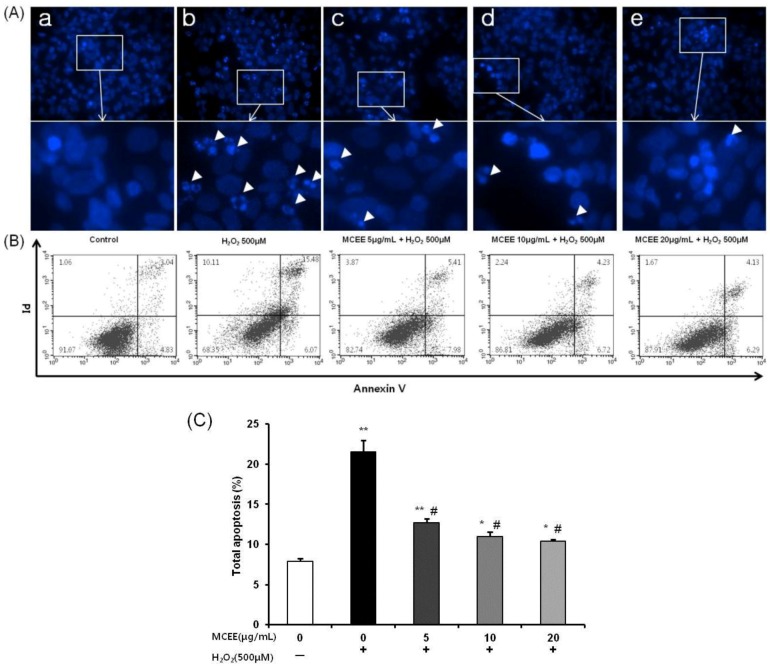
Effects of MCEE on apoptosis in H_2_O_2_-induced SK-N-MC cells. (**A**) Morphological changes of nuclear chromatin by Hoechst 33342 staining were observed using a fluorescence microscope. Hoechst dye stained both apoptotic nuclei (arrowheads) and condensed nuclei. (**a**) Control, (**b**) H_2_O_2_ 500 μM, (**c**) MCEE 5 μg/mL + H_2_O_2_ 500 μM, (**d**) MCEE 10 μg/mL + H_2_O_2_ 500 μM, (**e**) MCEE 20 μg/mL + H_2_O_2_ 500 μM (**B**) Annexin V/PI staining by flow cytometry. Quadrant analysis of the fluorescence characteristics of four panels: viable cells on the lower left, Annexin V(−)/PI(−); necrotic cells on the upper left, Annexin V(−)/PI(+); late apoptotic cells on the upper right, Annexin V(+)/PI(+) and early apoptotic cells on the lower right, Annexin V(+)/PI(−). (**C**) Quantitative analysis of the bar graphs showed the percentage of total apoptotic cells. Data are shown as means ± S.D. (*n* = 3). * *p* < 0.05; ** *p* < 0.01 compared with the untreated control cells, # *p* < 0.05 compared with only H_2_O_2_-treated cells.

To verify whether MCEE attenuated H_2_O_2_-induced apoptosis, Hoechst 33342 staining and Annexin V-PI double staining were performed. As shown in Figure 5A, the H_2_O_2_-stimulated group revealed apoptotic and condensed nuclear characteristics. However, these were dramatically ameliorated in the SK-N-MC cells by pretreatment with MCEE. The possible nuclear-protective effect of MCEE was confirmed by flow-cytometric analysis with the Annexin V-PI double-staining assay. As shown in Figure 5B,C, treating cells with H_2_O_2_ (500 μM) for 4 h resulted in an increase in the percentage of cells in total apoptosis to 20.54% ± 1.43% compared with the untreated control cells (8.14% ± 0.38%). However, pretreatment with MCEE (5, 10, and 20 μg/mL) for 24 h before exposure to H_2_O_2_ reduced the total apoptotic cell population in a dose-dependent manner to 13.04% ± 0.49%, 11.35% ± 0.57%, and 10.54% ± 0.18%, respectively.

### 3.5. MCEE Ameliorates H_2_O_2_-Induced Mitochondrial Membrane Potential (MMP, Δψ_m_)

As the change of ΔΨ_m_ is considered to be closely related to apoptosis, loss of ΔΨ_m_ is used as an indicator of apoptosis [34]. JC-1 dye and flow cytometric analysis were used to assess the change of ΔΨ_m_ during apoptosis induced by H_2_O_2_ and the protection of MCEE in SK-N-MC cells.

**Figure 6:**
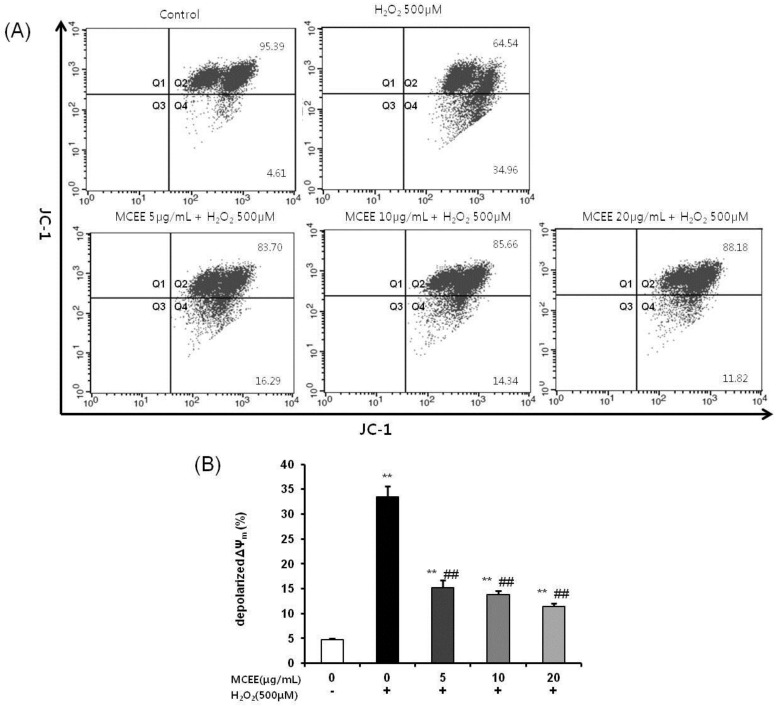
Effect of MCEE on the mitochondrial membrane potential (ΔΨ_m_) in SK-N-MC cells. Cells were pretreated with MCEE for 24 h and then treated with H_2_O_2_ for 4 h. JC-1 staining was analyzed by flow cytometry. ΔΨ_m_ was measured using JC-1 fluorescence dye by a flow cytometer. (**A**) Quadrant analysis of the fluorescence characteristics of four panels: Q2, red fluorescence +/green fluorescence +, polarized ΔΨ_m_; Q4, red fluorescence −/green fluorescence +, depolarized ΔΨ_m_. (**B**) Quantitative analysis of the bar graphs showed the percentage of depolarized ΔΨ_m_. Data are shown as means ± S.D. (*n* = 3). ** *p* < 0.01 compared with the untreated control cells, ## *p* < 0.01 compared with only H_2_O_2_-treated cells. JC-1, 5,5’,6,6’-tetrachloro-1,1’,3,3’-tetraethyl-enzimidazolcarbocyanine iodide.

In normal cells with a polarized ΔΨ_m_, JC-1 aggregated with healthy mitochondria and exhibited red fluorescence. When ΔΨ_m_ depolarized during apoptosis, JC-1 existed in the monomer form on the cytoplasm and exhibited green fluorescence. As shown in Figure 6A, JC-1 fluorescence was observed in the red and green channels (Q2) in the control cells, while there was a significant increase in the number of cells with lowered red fluorescence (Q4), which meant that a depolarized ΔΨ_m_ occurred in the H_2_O_2_ treated cells (33.48% ± 2.09%). Pretreatment with MCEE (5, 10, and 20 μg/mL) markedly reduced the number of cells with depolarized ΔΨ_m_ to 15.25% ± 1.48%, 13.77% ± 0.81%, and 11.36% ± 0.66%, respectively.

### 3.6. MCEE Modulated the Expression of Apoptosis-Related Proteins Expression Induced by H_2_O_2_

**Figure 7:**
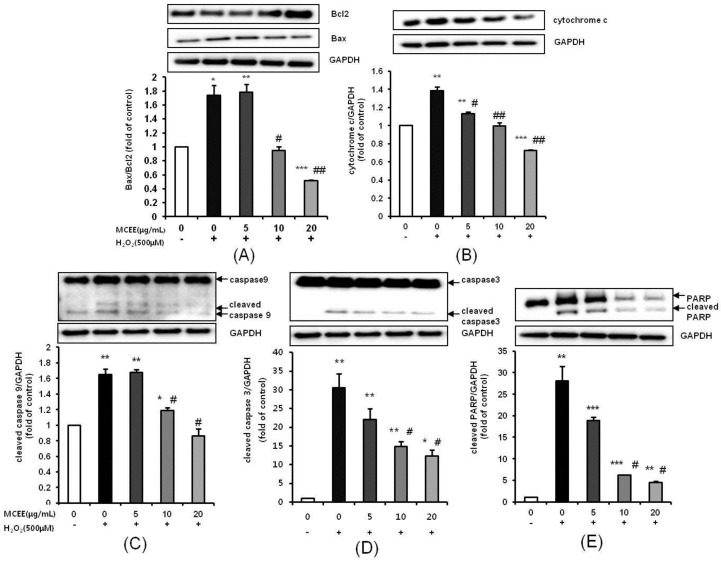
Neuroprotective effects of MCEE on the apoptosis-related proteins in H_2_O_2_-treated SK-N-MC cells. Cells were pretreated with the MCEE for 24 h and then treated with H_2_O_2_ for 4 h. (**A**) Bax, Bcl2; (**B**) cytochrome c; (**C**) caspase-9, cleaved caspase-9; (**D**) caspase-3, cleaved caspase-3; and (**E**) PARP, cleaved PARP protein expression was confirmed by Western blot analysis. The normalization of cytochrome c, cleaved caspase-9, cleaved caspase-3, and cleaved PARP used GAPDH. The results are expressed as means ± S.D. (*n* = 3). * *p* < 0.05; ** *p* < 0.01; *** *p* < 0.001 compared with the untreated control cells, # *p* < 0.05; ## *p* < 0.01 compared with only H_2_O_2_-treated cells. PARP, poly (ADP-ribose) polymerase.

We then investigated the apoptosis-related protein expression in H_2_O_2_-induced SK-N-MC cells in the presence or absence of MCEE. After exposure to 500 μM H_2_O_2_, the ratio of Bax:Bcl-2 and the expression of cytochrome c were increased (Figure 7A,B). These trends were reversed by MCEE in a dose-dependent manner. Consistently, the upregulation of cleaved caspase-9, caspase-3, and PARP, which are pro-apoptotic markers, was significantly suppressed in a dose-dependent manner by pre-treatment with MCEE (Figure 7C–E).

### 3.7. MCEE Attenuates the H_2_O_2_-Induced Activation of the JNK, p38, and ERK1/2 MAPK Signaling Pathway

We further examined the effects of MCEE on the mitogen-activated protein kinase signaling (MAPKs) pathway, such as p38, JNK, and ERK, which are known to be involved in oxidative stress-induced cell death [35]. As shown in Figure 7, the expression levels of p-JNK, p-p38, and p-ERK 1/2 were significantly increased after treating the cells with H_2_O_2_. Pretreatment of the cells with MCEE significantly inhibited the H_2_O_2_-induced phosphorylation of JNK, p38, and ERK 1/2. These results collectively demonstrate that: (1) MCEE blocked the triggering of the mitochondria-dependent apoptosis pathway by H_2_O_2_; and (2) MCEE reduced cell death via direct inactivation of the MAPKs pathway.

## 4. Discussion and Conclusions

**Figure 8:**
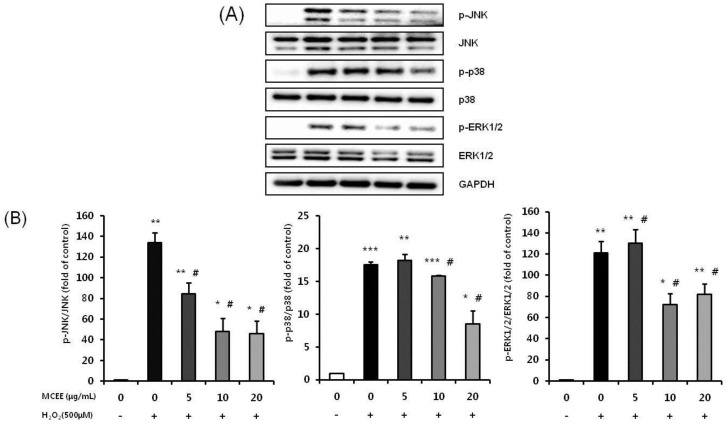
Neuroprotective effects of MCEE on the MAPKs pathway in H_2_O_2_-induced SK-N-MC cells. Cells were pretreated with the MCEE for 24 h and then treated with H_2_O_2_ for 4 h. JNK, p-JNK, p38, p-p38, ERK 1/2, and p-ERK 1/2 protein expression levels were confirmed by Western blot analysis. (**A**) The proteins expression by Western blot analysis. (**B**) Quantitative analysis of the bar graphs showed the densities of the protein bands. Data are shown as means ± S.D. (*n* = 3). * *p* < 0.05; ** *p* < 0.01; *** *p* < 0.001 compared with the untreated control cells, # *p* < 0.05 compared with only H_2_O_2_-treated cells.

Oxidative stress due to ROS plays critical roles in the pathophysiology of neurodegenerative diseases that are related to the molecular mechanisms of neuronal cell death, such as exotoxicity, free radical damage, and neuroinflammation [5,36]. This study hypothesized that *Momordica charantia* (MC) extract protects neuronal cells against H_2_O_2_-induced oxidative stress. Here, we demonstrated that an ethanol extract of MC reduces neurotoxicity through the attenuation of ROS levels (Figure 1 and Figure 2), the enhancement of the intracellular antioxidant system (Figure 3 and Figure 4), and the inhibition of apoptosis by suppressing the mitochondrial-dependent intrinsic (Figure 5, Figure 6 and Figure 7) and MAPKs pathway (Figure 8) in SK-N-MC cells. To our knowledge, this is the first study to report that MC has potential as a promising neuroprotective agent through its antioxidant and anti-apoptotic properties.

We investigated the previously unappreciated value of MC as a neuroprotective dietary compound. The reasons for this were as follows: (1) MC is cultivated all over the world; (2) patients with chronic diseases, such as diabetes, which are common in the elderly population, usually ingest MC to improve their disease state; and (3) there have been many studies on the health benefit of MC based on its antioxidant-related effects. It has been reported that MC extract and juice effectively protect against hepatotoxicity [24], enhance memory function [37], and attenuate cerebral ischemia/reperfusion injury [38]. In our previous study, the MC ethanol extract (MCEE) showed a high total polyphenol content (TPC) and total flavonoid content (TFC), as well as an excellent DPPH radical scavenging ability. Therefore, we decided that it was appropriate to confirm the protective effect on the oxidative stress of MCEE in neuronal cells.

In this study, we noticed that MCEE pretreatment reversed H_2_O_2_-induced cell damage, which was confirmed by reduced cell viability and increased intracellular LDH release (Figure 1). Hydrogen peroxide (H_2_O_2_), the most important ROS contributor, is easily accessible via the cell membrane and can be converted to the highly reactive hydroxyl radical (•OH) via the Fenton reaction [39]. ROS can oxidize essential biological macromolecules such as lipids, proteins, and DNA, and induce mitochondrial damage and induce cell death through several pathways in a variety of cells, including neuronal cells [13,32,40]. Figure 2 shows that MCEE pretreatment reversed H_2_O_2_-induced cell damage through the inhibition of intracellular ROS production. In agreement with our results, several studies have reported that various natural product extracts ameliorate the negative changes of cell viability, LDH release, and intracellular ROS level by H_2_O_2_ [10,11,12,13,15]. Furthermore, the reduction in oxidative stress by various natural products is associated with enhanced antioxidant defense, inhibited apoptosis, and regulation of the MAPKs and AKT/GSK-3b pathway. Thus, it may be reasonable to assume that these beneficial biological effects might have neuroprotective effects. Here, we tried to validate the mechanism of the above relationship on the neuroprotective effects of MCEE.

One possibility is that the neuroprotection of MCEE may be related to the antioxidant activity. Our results demonstrated that MCEE pretreatment increased the SOD activity and GSH level, while it decreased the MDA level (Figure 3). This implies that MCEE enhanced ROS scavenging activity through upregulation of the antioxidant system and consequently, alleviated the oxidative damage induced by H_2_O_2_. Moreover, MCEE pretreatment significantly increased both mRNA and protein level HO-1 expression (Figure 4). HO-1 is an antioxidant and phase II detoxification enzyme that plays an important role in maintaining antioxidant homeostasis. It is present in most cells and tissues and is a key molecule for the adaptive cellular response in oxidative stress conditions [33,41]. Our results indicated that MCEE pretreatment positively affected the maintenance of antioxidant homeostasis through the upregulation of HO-1. Taken together, we suggest that the neuroprotective effect of MCEE is related to the improvement of cellular antioxidant status. Several in vivo and in vitro studies have reported that MC is a good source of antioxidants because it contains bioactive components, such as polysaccharides, polyphenols, flavonoids, saponins, and terpenoids [19,22]. MC is rich in flavonoids and phenolic compounds, including gallic acid, catechin, syringic acid, quinic acid, and caffeic acid [42,43,44]. The flavonoid and phenolic compounds that are major bioactive components of the natural products are widely known for their excellent antioxidant effects. In addition, the polysaccharides from MC ameliorate oxidative stress through the inhibition of the NK-κB signaling pathway [45]. Moreover, terpenoids induce Nrf2 through the Michael reaction of reactive cysteine residues on the Keap1 protein. As Nrf2 induces the activation of HO-1, various terpenoids have been reported to possess antioxidant and protective effects [46,47]. Therefore, we suggest that MCEE might enhance the cellular antioxidant condition through the synergic effects of the above-mentioned bioactive components. In future studies, we will analyze the types and contents of the bioactive components of MCEE. Additionally, we will evaluate the possibility of the regulational action of MCEE on the Nrf2 signaling pathway that can regulate a lot of genes, which encode cytoprotective proteins involved in major diseases such as inflammation, cancer, and neurodegenerative disease.

Subsequently, another possibility of the neuroprotective mechanism of MCEE is its anti-apoptosis activity as oxidative stress has been reported to trigger mitochondrial-dependent cell death cascades [16,31]. Mitochondrial-dependent cell death is one of the three pathways that cause apoptosis through the action of cysteine aspartyl-specific proteases (caspases), which corresponds to the intrinsic apoptosis pathway. When mitochondria are damaged by ROS, mitochondrial outer membrane permeabilization occurs and MMP (ΔΨ_m_) collapses. When the level of depolarized ΔΨ_m_ increases, the cytochrome c present in the mitochondrial intermembrane space is released into the cytoplasm, which activates caspase-9 by binding to apoptosis protease activating factor (APAF)-1. Activated caspase-9 activates caspase-3 and results in apoptotic cell death [8,13,34,48]. The mitochondria-dependent cell death cascade is regulated by the Bcl2 family. The balance between the anti-apoptotic protein, Bcl2, and the pro-apoptotic protein, Bax, is considered crucial for cell survival and death. An increased Bax/Bcl2 ratio induces cytochrome c release from the mitochondria into the cytoplasm and activates the intrinsic apoptotic pathway [13,31]. Our data have shown that MCEE reduced upregulation of apoptosis (Figure 5) and depolarized ΔΨ_m_ (Figure 6) by H_2_O_2_. In addition, the expression of apoptosis-related proteins such as Bax, Bcl2, cytochrome c, and cleaved caspase-9, caspase-3, and PARP was significantly modulated by MCEE (Figure 7). This implies that MCEE inhibits the intrinsic apoptosis pathway by alleviating mitochondrial dysfunction by oxidative stress. Several studies have supported the notion that various natural product extracts inhibit apoptosis induced by H_2_O_2_ [32], rotenone [8], and glutamate [11] in human and/or rat neuronal cell lines and this has been confirmed by the increased depolarized ΔΨm, Bax/Bcl2 ratio, and cytochrome c expression and activated caspases. It has been suggested that the natural product extracts used in each study protect cells through inhibition of the mitochondrial-dependent apoptosis pathway. There have been many reports that the anti-apoptotic effect of natural products occurs through their bioactive components. Polyphenols, such as quercetin, resveratrol, and rosmarinic acid, the main bioactive components of natural products, inhibit the intrinsic apoptosis pathway by alleviating the destabilization of mitochondria membranes caused by mitochondrial dysfunction [49,50]. Asiatic acid (AA), which was triterpene extracted from *Centella asiatica*, protects human neuronal cells against rotenone-induced mitochondria dysfunction and oxidative stress-mediated apoptosis [16]. In addition, Liu et al. [51] demonstrated that polysaccharides of *Cordyceps* protect HL-7702 cells against H_2_O_2_-induced mitochondrial dysfunction, such as decreased MMP, reduced ATP, and the promotion of cytochrome release. Polysaccharides from MC were reported to inhibit apoptosis by the modulation of apoptotic markers such as caspase 3, Bax, and Bcl2 in the myocardial infarction model [45] and by the inactivation of JNK3/cytochrome c/caspase-3 in the cerebral ischemia/reperfusion injury model [52]. These studies supported the notion that the anti-apoptosis activity of MCEE, including various ingredients such as polyphenols, triterpenes, and polysaccharides, might be due to the blockage of mitochondrial-dependent cell death cascades by inhibiting mitochondrial cytochrome c-release by mitochondria membrane stabilization and modulating the apoptosis-related protein expression.

Additionally, we investigated whether MCEE affects the MAPKs pathway through its anti-apoptosis activity. Our results showed that MCEE pretreatment significantly inhibited the phosphorylation of p38, JNK, and the ERK1/2 MAPKs pathway induced by H_2_O_2_ (Figure 8). This implies that MCEE could inhibit apoptosis by the inactivation of the MAPKs pathway. Numerous studies have reported that the activation of the MAPKs pathway by oxidative stress induces the apoptotic death of neuronal cells that are the major pathology in many neurodegenerative diseases [10,53,54]. Therefore, measuring the MAPKs pathway could be important for the screening of therapeutic agents with neuroprotective effects [11,55]. Several studies have reported that natural product extracts could inhibit oxidative stress-induced apoptosis through decreasing the Bax/Bcl2 ratio and inhibiting the expression level of apoptosis-related proteins, such as cytochrome c, caspase-3, and PARP, as well as the phosphorylated p38, JNK, and ERK1/2 MAPKs [10,11]. Park et al. [15] reported that the ethanol extract of *Liriope platyphylla* inhibits the expression of cleaved caspase-3 and PARP by modulating p38 MAPKs pathway activation in SH-SY5Y cells. They suggested that the extract used in each study has the potential to be an agent for the treatment of NDs. Thus, it may be reasonable to assume that MCEE could be beneficial in the treatment of NDs. However, further study is required to elucidate mechanical evidence on the anti-apoptotic mechanism of MCEE. Thus, we will examine the role of MCEE on the ROS-mediated AKT/ASK/MAPKs pathway and also investigate the possible action of MCEE on the cell cycle.

Despite the many reports that natural products have health benefits, there are always questions raised in the case of experimental research, for example, the suitability of the treatment concentration to the experimental conditions; and the dietary consumption required to expect equivalent effects in humans. In a study by Shobha et al. [17], 2 μg/mL of MC in 50% ethanol extracts induced adipogenesis inhibition and the promotion of adipolysis in 3T3-L1 pre-adipocyte cells. Gong et al. [52] reported that polysaccharides of MC inhibited apoptosis from primary cultured hippocampal neurons at concentrations greater than 10 μg/mL. As our study used an in vitro model that induced cell damage using H_2_O_2_, a major ROS contributor, it required an excellent agent to act as a radical scavenger to protect cells from oxidative stress. Therefore, we assumed that MCEE would be effective at higher concentrations than those inhibiting adipogenesis. Furthermore, the treatment concentration was similar to or higher in the study of natural product ethanol extracts reported to have a neuroprotective effect than our concentration of MCEE [11,15,40]. In particular, the total phenolic content (TPC) of the *Arctium lappa* L. roots ethanol extract (EAE) [11] was detected as being 88.9 mg gallic acid equivalents/g extract. It was observed that the protective effects on glutamate-induced oxidative stress in PC12 cells occurred at a concentration of 20 μg/mL or more. Although the TPC of MCEE used in our study was 29.5 mg gallic acid equivalents/g extract lower than the EAE, the protective effects of MCEE against induced oxidative stress were effective at a lower concentration than the EAE. Thus, we used 5, 10, and 20 μg/mL of MCEE as treatment concentrations to assess the potency of the protective effect of MCEE, and this seemed appropriate. Deng et al. [24] reported that oral administration of 500 mg/kg MC water extract was the most effective to protect against liver injury in restraint-stressed mice. They stated that they would need to drink approximately 7.5 g of MC dried fruit to get an equivalent dose, considering the difference in the metabolic rates of mice and humans and the extract yield. The TPC of MCEE used in our study was about 2.5 times higher than their MC water extract (11.7 mg gallic acid equivalents/g extract). Thus, MCEE is also expected to have an effective antioxidant capacity in the experimental animal model. Considering this, further studies are necessary to confirm the protective effect of MCEE in the experimental animal model and to identify whether MCEE or its bioactive components affect the brain, the target tissue to treat NDs.

The human neuroblastoma SK-N-MC cells that were used in our study have been widely used to investigate the pathogenesis and molecular mechanism of NDs, including AD [1,30]. Additionally, the other human neuroblastoma cells, such as SH-SY5Y and SK-N-SH, are well-known as an in vitro neuronal cell model for studying the pathogenesis of PD, one of the NDs [56]. Although these are neuronal-like cells, these cell lines could not completely reflect the general character of the neuronal cells due to its cancerous origin. Therefore, additional verification may be required in normal neuronal cells using the primary culture of the cortical neuron or hippocampal neuron, or some animal models.

In conclusion, the present study observed that pretreatment of MCEE could protect neuronal cells from H_2_O_2_-induced oxidative stress. We identified that MCEE effectively alleviates the neuronal cell damage caused by intracellular oxidation/antioxidant imbalance and the pathway of cell death, such as apoptosis. Further studies should be conducted to determine the efficacy of MCEE in rodent models. Nevertheless, we believe that these discoveries support the potential benefit of MCEE as a novel neuroprotective agent which should be used in future clinical studies.
